# Cystobactamid off-target profiling reveals favorable safety, superoxide reduction, and SCARB1 inhibition in eukaryotes

**DOI:** 10.1038/s44386-025-00020-7

**Published:** 2025-08-06

**Authors:** Timo Risch, Benedikt Hellwinkel, Dietrich Mostert, Andreas M. Kany, Danny Solga, Tim Seedorf, Dominik Heimann, Jessica Hoppstädter, Daniel Kohnhäuser, Jil-Sophie Hilgers, Franziska Fries, Felix Deschner, Mark Brönstrup, Andreas Kirschning, Stephan A. Sieber, Thomas Pietschmann, Alexandra K. Kiemer, Jennifer Herrmann, Rolf Müller

**Affiliations:** 1 Helmholtz Institute for Pharmaceutical Research Saarland (HIPS), Helmholtz Centre for Infection Research (HZI), Saarland University, PharmaScienceHub (PSH), Campus E8.1, 66123 Saarbrücken, Germany; 2 German Center for Infection Research (DZIF), Inhoffenstraße 7, 38124 Braunschweig, Germany; 3 TWINCORE, Centre for Experimental and Clinical Infection Research, a joint venture between the Helmholtz Centre for Infection Research and the Hannover Medical School, Hannover, Germany; 4 Center for Functional Protein Assemblies (CPA), Department of Chemistry, Chair of Organic Chemistry II, Technical University of Munich, Ernst-Otto-Fischer-Straße 8, 85748 Garching, Germany; 5 Leibniz University Hannover, Institute of Organic Chemistry, Schneiderberg 1B, 30167 Hannover, Germany; 6 Helmholtz Centre for Infection Research (HZI), Inhoffenstraße 7, 38124 Braunschweig, Germany; 7 Department of Pharmacy, Pharmaceutical Biology, Saarland University, PharmaScienceHub (PSH), Campus C2 3, 66123 Saarbruecken, Germany; 8 Uppsala Biomedical Center (BMC), Uppsala University, Husargatan 3, 752 37 Uppsala, Sweden; 9 Cluster of Excellence RESIST (EXC 2155), Hannover Medical School, Hannover, Germany

**Keywords:** Drug safety, Pharmacology, Target identification, Toxicology

## Abstract

Antimicrobial resistance poses a fundamental global threat, necessitating new strategies for effective therapies. Cystobactamids, a class of antibacterial agents targeting bacterial gyrase and topoisomerase IV, represent a non-traditional chemical scaffold with broad-spectrum activity. For toxicological de-risking, we performed a comprehensive profiling on eukaryotic cells, focusing on cytotoxicity, genotoxicity, and mitochondrial toxicity, demonstrating cellular safety and superoxide scavenging properties. Studies in zebrafish embryos assessed developmental, cardiovascular, and hepatic toxicity, indicating a favorable in vivo safety profile. Metabolism studies revealed glucuronidation and amide bond hydrolysis as key pathways, whereby cystobactamid metabolic stability substantially improved by cobicistat co-treatment. Affinity-based protein profiling identified the cholesterol- and HCV-receptor scavenger receptor class B member 1 (SCARB1) as a primary eukaryotic off-target protein, with cystobactamids shown to inhibit SCARB1´s function, preventing hepatitis C virus pseudoparticle entry into cells. These findings suggest a high therapeutic potential for cystobactamids and highlight SCARB1 as a primary eukaryotic target.

## Introduction

Leading health institutions, including the European medicine agency (EMA) and the World Health Organization (WHO), call attention to antimicrobial resistance as an increasing global risk to patients and healthcare systems^1–4^ The misuse and overuse of antibiotics in humans and also in animals drive the emergence and spread of multidrug-resistant (MDR) bacteria. At the same time, the number of future treatment options using novel antibiotics with innovative structures and mechanisms of action is insufficient^4,5^. The development of previously identified cystobactamids (CYS) might help to fill this gap, as they represent promising broad-spectrum antibiotics comprising a novel scaffold^6^.

CYS are natural products derived from *Cystobacter* and *Myxococcus* spp^7^. They exhibit antibacterial activity against a broad spectrum of clinically relevant Gram-negative and Gram-positive bacteria, including MDR isolates of *Acinetobacter baumannii*, *Enterococcus faecalis*, *Streptococcus pneumoniae*, *Staphylococcus aureus* and *Escherichia coli*^6,7^. CYS act through a new mechanism of action by inhibiting bacterial type IIa topoisomerases (gyrase and topoisomerase IV)^6^. A dual mode of binding for the structurally related albicidin and CYS was recently described with one part of the molecule blocking the gyrase dimer interface and the other end intercalating between cleaved DNA fragments, hence preventing DNA religation^8^. Importantly, CYS and the structurally related compound classes of albicidin and coralmycin represent a novel chemical scaffold consisting of *para*-nitrobenzoic acid and multiple *para*-aminobenzoic acid units connected through an amino acid linker^9,10^. The novel structure, the lack of cross-resistance with commercially used drugs and their new mechanism of action contribute to the observed resistance-breaking properties within clinical isolates of MDR pathogens^6,7,11–13^. Furthermore, CYS were shown to have a low frequency of resistance^13^, which is of utmost importance for the wide and sustainable use as an antibiotic. Several total syntheses of CYS were established and modified to successfully yield more than hundred derivatives^7,11,12,14,15^. This allowed for large-scale production and structural modifications to derive structure-activity and structure-property relationships for optimization of potency, antibacterial spectrum coverage, on-target activity, and physicochemical properties^14,15^.

To ensure efficacy and safety, pharmacological and toxicological properties need to be determined before entering clinical studies. In fact, leading causes for failure of drug candidates in the drug development process are lack of efficacy (52%) or an unfavorable safety profile (24%) (2013–2015)^16,17^. Drug approval requires three key properties, namely high-quality standards during the production procedure, efficacy against the target disease or symptoms, and a favorable safety profile^18^. Overall, the benefit of using a certain drug must exceed the risk of serious adverse effects^19^. Adverse or side effects include innumerable symptoms, which can range from non-critical effects like dizziness or headaches to life-threatening events such as liver damage or arrhythmia^20,21^.

In the presented study, we evaluated the cyto-, geno- and mitotoxicity of CYS derivatives CN-861-2, CN-DM-861 and Cysto-180 using in vitro cell culture models, and we investigated more complex organotoxicity with regards to general developmental, cardio- and hepatotoxicity by in vivo zebrafish embryo models. Furthermore, metabolic pathways and biotransformation of CYS were investigated in vitro, and a strategy to improve their in vivo exposure was proposed. Moreover, eukaryotic off-target proteins of CYS were identified and analyzed on a molecular level by affinity-based proteome profiling and functional inhibition assays, surprisingly uncovering yet unexplored potential therapeutic areas for CYS outside their use as antibacterial agents.

## Results and discussion

### CYS demonstrates general safety in cytotoxicity and genotoxicity assays, with a mild effect on uncoupling the mitochondrial electron transfer chain (ETC)

**Fig. 1:**
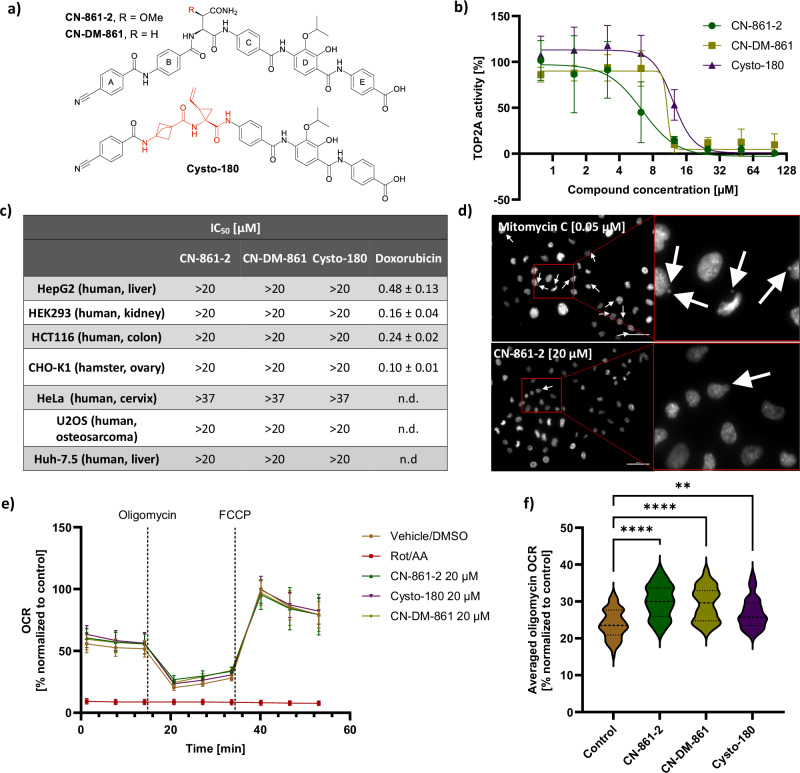
CYS demonstrates general safety in cytotoxicity and genotoxicity assays, with a mild effect on uncoupling the mitochondrial electron transfer chain (ETC). **a** Chemical structures of tested CYS derivatives CN-861-2 (top), CN-DM-861 (top) and Cysto-180 (bottom) in comparison. N-terminal rings A and B are connected via an amino-acid linker with C-terminal rings C to E. CN-861-2 served as reference in this study and structural differences between derivatives are marked in red. **b** Cystobactamids show concentration-dependent inhibition of the human topoisomerase IIα. Data are represented as mean values with standard deviation. IC_50_ was evaluated using non-linear regression (*n* = 3). **c** CYS derivatives showed no harmful effect on cell viability on human and non-human immortalized cell lines in their solubility range (*n* = 3) (n.d., not determined). **d** CYS-treated cells showed micronucleus formation comparable to the DMSO control (see also Supplementary Fig. 1), while mitomycin C-treated cells showed extensive micronucleus formation. (nuclei colored in white, white arrows indicate micronuclei, scale bar = 50 µm) (*n* = 3). **e** Seahorse mitotoxicity assay revealed a slight uncoupling effect of CYS, determined as an increased oxygen consumption rate (OCR) following oligomycin addition. A combination of the complex I inhibitor rotenone (Rot) and the complex III inhibitor antimycin A (AA) was used as a positive control. Data are represented as mean values with standard deviation (*n* = 3 × 6 wells). **f** Normalized mean values (% normalized to max OCR of control group) with standard deviations are shown (*n* = 3 × 6). Statistically significant differences of the oligomycin OCR were analyzed by ordinary one-way ANOVA with multiple comparisons to the DMSO-treated control group. (**p < 0.01; ****p < 0.0001). A violin plot is used for data representation.

Three CYS derivatives (CN-861-2, CN-DM-861 and Cysto-180; Fig. 1a) were chosen to further investigate properties of the class with respect to their biological activity on eukaryotic cells. The selected derivatives comprise early stage frontrunner molecules with improved antibacterial properties (CN-861-2 and CN-DM-861^15^; the former served as the main reference compound in this study due to its availability) and a further improved derivative (Cysto-180^13^).

Since CYS are bacterial topoisomerase II inhibitors and thus, affect DNA synthesis and repair^6^, an obvious potential molecular off-target of CYS is the human DNA topoisomerase IIα (TOP2A). Half inhibitory concentrations (IC_50_) of 6.26–12.22 µM (Fig. 1b) against TOP2A were determined (CN-861-2 IC_50_ = 6.26 ± 4.69 µM, CN-DM-861 IC_50_ = 10.83 ± 2.38 µM, Cysto-180 IC_50_ = 12.22 ± 0.63 µM), which range far beyond (~100 fold) previously observed minimal inhibitory concentrations (MIC) of CYS for their target pathogens as well as identified IC_50_ on *E. coli* gyrase (CN-DM-861 IC_50_ = 0.08 µM)^13,15^.

Cell culture models are widely used to determine the cytotoxicity of compounds and to initially assess whether molecules interact with fundamental cellular functions affecting cell division and viability^22^. Interestingly, despite their inhibitory effect on the isolated TOP2A activity, CYS did not show any reduction of cell viability in their tested solubility range (≤20 µM) for any tested human and non-human cell line (Fig. 1c). Cysto-180 was more soluble than CN-861-2 and CN-DM-861 and could be tested at higher concentrations. Cysto-180 did not exert cytotoxic effects in Huh-7.5 or CHO-K1 cells with an IC_50_ > 100 µM, underlining the safety of this compound class in the cell viability assay. False negatives resulting from CYS binding to fetal bovine serum (FBS) present in the cell culture media can be excluded, since we observed only a negligible shift in MIC (2-fold) for the tested reference strain *E. coli* ATCC25922 when supplementing the bacterial growth media (cation-adjusted Mueller-Hinton broth) with 10% (*v/v*) FBS.

In order to assess whether topoisomerase inhibition or previously observed minor groove binding would translate into genotoxicity in a cellular context, a micronucleus assay was performed^8,11^. The DNA cross-linker mitomycin C (0.05 µM), the intercalator doxorubicin (0.05 µM) and the topoisomerase II poison etoposide (0.25 µM) showed a high amount of nuclear bud/micronuclei formation. Interestingly, etoposide as TOP2 poison showed substantial micronuclei formation far below its IC_50_ for TOP2A (46.3 µM, determined by Inspiralis). Cells treated with CYS at concentrations exceeding their TOP2A IC_50_ (20 µM, 100 µM for Cysto-180) also exhibit some micronuclei/nuclear bud formation, but the extent was comparable to that observed in the DMSO control (Fig. 1d, Supplementary Fig. 1). Despite the topoisomerase inhibition observed on the isolated protein, these results suggest a relatively safe genotoxic profile in a whole cell environment, potentially explainable by poor passive eukaryotic membrane permeability of tested CYS derivatives. Nevertheless, further de-risking measures in the development of CYS are highly recommended, as the possibility of a genotoxic effect cannot be excluded^23,24^.

Given the known risk of quinolone antibiotics interfering with the mitochondrial topoisomerases and ETC-proteins, thereby harming mitochondrial functions^25,26^, CYS were further tested in a Seahorse XF Mito Tox Assay (Agilent) to evaluate mitotoxic risks. This assay measures the oxygen consumption rate (OCR) of HepG2 cells treated with the test compounds. During the assay, oligomycin is added as an inhibitor of the oxidative phosphorylation, thus oversaturating the electrochemical gradient at the inner mitochondrial membrane and thereby causing a decrease in the OCR. Subsequently, carbonyl cyanide-p-trifluoromethoxyphenylhydrazone (FCCP) (ionophore) addition leads to an uncoupling of the ETC from the oxidative phosphorylation, thereby increasing the OCR. Inhibition of the oxidative phosphorylation can be seen as a decrease of the starting OCR, whereas uncoupling is defined as an increase in OCR after oligomycin addition. Furthermore, a reduction of the starting OCR and after FCCP addition indicates an inhibition of the ETC caused by the test compound. CYS showed neither inhibition of the oxidative phosphorylation, nor of the ETC due to reduced oxygen consumption at the starting conditions or after FCCP addition, respectively^27^. However, CYS showed a slight but significant uncoupling of the ETC from the oxidative phosphorylation after oligomycin addition with an uncoupling mitochondrial toxicity index (MTI) of 13–17% at their highest soluble concentrations (Fig. 1e, f). This indicates that CYS as lipophilic weak acids might act as mitochondrial protonophores, shuttling protons across the inner mitochondrial membrane^28^. CYS are likely to be present in a neutral charge form in the acidic environment of the mitochondrial intermembrane space. This protonation could enhance their membrane migration into the mitochondrial matrix, where the carboxylic acid group is subsequently deprotonated. This process might partially restore the electrochemical gradient and thereby the functionality of the ETC. However, this slight uncoupling did not seem to have a direct harmful effect on cell growth and viability as shown above.

### CYS treatment resulted in a significant reduction of superoxide radical formation

It has been shown that anti-infective agents frequently influence mitochondrial function^25^. To investigate potential mitochondrial toxicity caused by increased levels of reactive oxygen species (ROS) due to interruption of the ETC upon CYS treatment, superoxide radical (O_2_^•-^) production was examined using MitoSOX Red. ROS are byproducts of various essential biological functions and are involved in cell homeostasis and signaling. However, an excess of ROS due to cellular stress, (UV-)irradiation or xenobiotics leads to oxidation of biological components such as lipids, proteins and DNA, which ultimately interrupts their physiological function^29^. It has been shown that an increase in ROS in vivo can lead to e.g., heart or liver failure with increased mortality, as seen e.g., for the cancer chemotherapeutic class of anthracyclines^30–32^. The superoxide radical is the precursor of most ROS and thus represents an indicator used for the quantification of ROS formation.

**Fig. 2:**
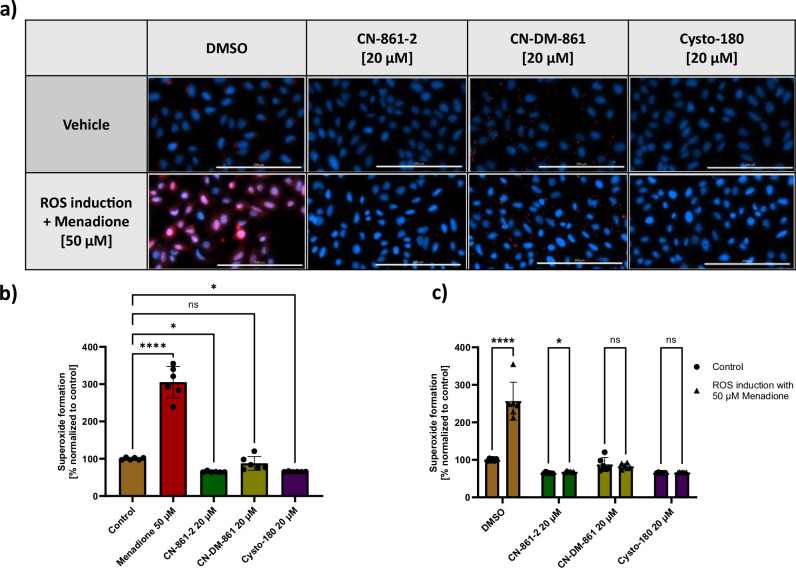
CYS treatment resulted in a significant reduction of superoxide radical formation. **a** Superoxide formation in U-2 OS cells (indicated by MitoSOX-red fluorescence (pseudocolor red), Hoechst-stained nuclei (pseudocolor blue)) (scale bars are set to 200 µm). **b** Cells showed significantly reduced basal levels of superoxide, when treated with CN-861-2 and Cysto-180. For CN-DM-861, some precipitation was observed and cells showed no ROS induction at the maximum soluble concentration. **c** Co-treatment with CYS suppresses menadione-induced ROS formation. Mean values with standard deviation are shown. Statistical significance was analyzed by ordinary one-way ANOVA with multiple comparison to the control group (A) and multiple unpaired two-stage step-up *t* test (B) (*n* = 6) (ns non-significant; *p < 0.05; ****p < 0.0001).

When comparing the superoxide formation of the DMSO control with the CYS-treated cells, there was no increase observable. To our surprise, a decrease of superoxide radical levels was observed in CN-861-2 and Cysto-180-treated samples compared to the control group. We also noticed precipitation of CN-DM-861, probably contributing to increased variation in measured fluorescence intensity caused by light scattering at the particles (Fig. 2a top row, Fig. 2b). In order to investigate whether CYS are able to intercept superoxide radicals, ROS formation via treatment with the redox-cycler menadione was induced and cells were co-treated with CYS^33^. Indeed, in the presence of CN-DM-861 and Cysto-180 no significant increase of superoxide was observed. CN-861-2 co-treated cells exhibited a slight yet statistically significant increase in superoxide production, though the induction remained considerably lower compared to the menadione control (Fig. 2a bottom row, Fig. 2c).

Nevertheless, some minor morphological abnormalities were also observed in CYS co-treated cells, indicating that a comprehensive ROS-protection was not achieved. However, these results show that CYS are able to counteract superoxide radical formation induced by menadione, and suggest that CYS generally prevent ROS formation to a certain extent. This effect might partially be explained by the observed slight uncoupling of the ETC. It was previously reported that mild uncoupling by mitochondrial uncoupling proteins reduces superoxide formation due to a slight proton leakage^34^. This proton leakage potentially decreases the transfer of excess electrons in the ETC to oxygen (electron leakage), which would subsequently lead to superoxide formation^35^. Furthermore, the structure of CYS could additionally serve as a radical scavenger due to radical resonance stabilization captured in its aromatic moieties^36^. These findings indicate that CYS do not induce ROS formation but serve as protective agents against oxidative cell stress.

### CYS are metabolized in hepatocytes by amide bond hydrolysis and glucuronidation, which can be suppressed by cobicistat supplementation

Investigation of the in vitro drug metabolism and pharmacokinetic (DMPK) properties of potential new drugs is a crucial aspect of their pharmacological and toxicological assessment that is essential for evaluating efficacy and safety profiles. The characterization of new molecules comprises e.g., plasma or metabolic half-life and thereby the drug’s ability to reach sufficient exposure in vivo in relevant compartments. In addition, the metabolic pathway of a compound can guide compound optimization and has a potential impact on toxicological properties, e.g., by formation of reactive or toxic intermediates, or by depleting detoxifying agents like glutathione^37,38^.

In vitro evaluations revealed metabolic stability of the tested CYS in mouse plasma (*t*_1/2_ > 240 min). In mouse liver microsomes (MLM), some turnover was observed with slight differences between the derivatives. Cysto-180 and CN-861-2 were stable with *t*_1/2_ > 120 min and 81% and 59% of parent compound remaining after 120 min, respectively, while CN-DM-861 had a half-life of 89 min (Table 1). When tested in murine hepatocytes containing the full complement of phase I and phase II drug metabolizing enzymes, degradation was observed for CN-DM-861 (*t*_1/2_ 94 ± 15 min), and Cysto-180 (*t*_1/2_ 41 ± 16 min), while CN-861-2 was stable for over >3 h (Table 1). These values are encouraging, particularly for CN-DM-861 and CN-861-2, and the rather low rate of metabolic clearance of CN-DM-861 is in line with the observed renal excretion of non-metabolized compound in mice^15^. Still, we were interested in a qualitative assessment of the metabolic pathways of CYS. The metabolites of CN-DM-861 and the less stable Cysto-180 in mouse hepatocytes were identified with the aim of generating information guiding further compound optimization towards reduced clearance. Additionally, this information is helpful for future de-risking in view of potentially toxic metabolites. These studies revealed amide bond hydrolysis and glucuronidation of the parent compound as the major metabolic pathways (Supplementary Figs. 2–5). In particular, the amide bond between ring C and ring D appeared to be metabolically labile.

**Table 1:** In vitro metabolism studies of CYS derivatives including mouse liver microsomal (MLM) and mouse hepatocyte half-life (*t*_1/2_), intrinsic clearance (Cl_int_) and plasma stability testing as well as plasma protein binding (PPB) (*n* ≥ 2)

Code	MLM *t*_1/2_ [min]/Cl_int_ [µL/mg/min]	Mouse Hepatocytes *t*_1/2_ [min]/Cl_int_ [µL/mg/10^6^ cells]	Mouse plasma *t*_1/2_ [min]	Mouse PPB [%]
**CN-861-2**	>120 / < 11.6^a^	>180 / < 5.1	>240	99.63 ± 0.16
CN-DM-861	89.0 ± 1.3 / 15.6 ± 0.2	94 ± 15 / 10 ± 2	>240	99.85 ± 0.08
Cysto-180	>120 / < 11.6^b^	41 ± 16 / 26 ± 12	>240	95.9 ± 2.4

^a^59 ± 11% remaining after 2 h

^b^81 ± 10% remaining after 2 h^1^

Exploring an alternative to chemical optimization with respect to these findings, we assessed the possibility of reducing the turnover of CYS in vivo by reducing hepatic uptake. Towards this end, we investigated its metabolic stability in the presence of cobicistat. This drug is known as an OATP1B and CYP3A inhibitor and is used as a co-treatment with HIV therapeutics to enhance their metabolic stability in vivo^39,40^.

Indeed, supplementation substantially increased the metabolic stability of CN-DM-861 in mouse hepatocytes in a concentration-dependent manner. Whether the observed increase in CYS stability is solely related to reduced transport into hepatocytes via OATP inhibition or also influenced by reduced CYP-mediated amide cleavage^41^, cannot be fully answered by these assays. The fact that CN-DM-861 metabolism was also reduced by cobicistat in liver microsomes shows that in principle, reduction of CYS metabolism via CYP inhibition is feasible (Supplementary Fig. 6)^41^. In any case, the combination of CYS with cobicistat represents a promising strategy to enhance their metabolic stability and availability in vivo. However, verification in murine pharmacokinetic studies is required to determine whether a combination with cobicistat leads to improved in vivo stability.

All compounds showed high plasma protein binding (PPB ~ 96–100%, Table 1), which might raise an issue in vivo due to reduced availability of free drug. Only the free compound fraction is capable of interacting with its antimicrobial target and thus, capable of causing a pharmacological effect. However, previous experiments demonstrated the capability of CYS for being efficacious in vivo as assessed in various murine infection models^15,42^. It is also important to consider that partial PPB might even be beneficial for increased metabolic half-life and prolonged exposure at the site of infection.

### In vivo toxicity evaluation of CYS in zebrafish embryos revealed no abnormalities in development, cardiac function, or liver morphology

In vitro assays are sufficient in providing a basic understanding of the pharmacological properties of a new compound on a cellular level but can often not cover the complexity of a whole organism. Zebrafish embryos can be used as a more complex in vivo model e.g., for the early evaluation of potential (organo-)toxicity of a compound and its potential phase I and phase II metabolites^43–45^. In compliance with the 3 R principle on the reduction, refinement and replacement of animal experiments, we applied this model since zebrafish at early developmental stages (≤120 h post-fertilization, hpf) are not classified as animal models according to Directive 2010/63/EU^46^.

**Fig. 3:**
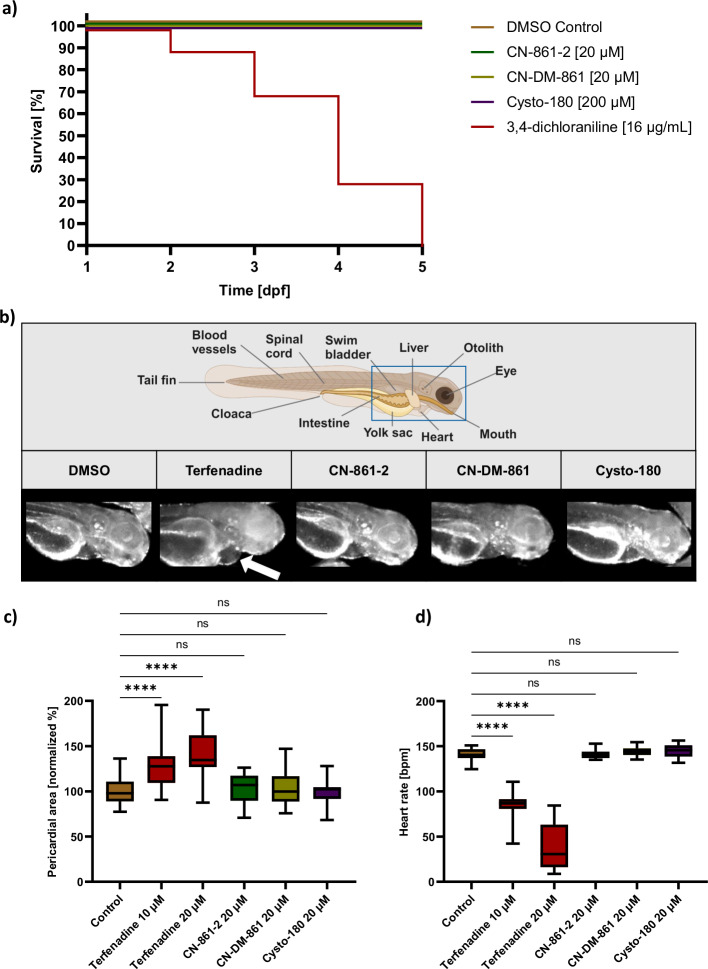
In vivo toxicity evaluation of CYS in zebrafish embryos revealed no abnormalities in development or cardiac function. **a** CYS showed no developmental in vivo toxicity. All CYS-treated zebrafish embryos survived and showed no abnormal development when exposed from 1 to 5 dpf. Maximum tolerated concentration of CYS was shown to be above their solubility limit (*n* = 20). **b** For assessment of cardiotoxicity, zebrafish embryos were treated from 2 to 3 dpf with no observable pathological cardiac morphology for CYS. For the positive control terfenadine, pericardial edemas were observable (white arrow) (Created in BioRender. Risch, T. (2025) https://BioRender.com/u43t186). **c** Statistical evaluation of the pericardial area showed no abnormalities for CYS treated embryos, but an increase in pericardial area after terfenadine treatment. **d** Statistical evaluation of the embryo´s heart rates showed no abnormalities after CYS treatment, but a decreased heart rate after treatment with the positive control terfenadine. Box plot (quartiles Q1 to Q3, including median) with whiskers (min to max) is shown. Statistical significance was analyzed by ordinary one-way ANOVA with multiple comparisons to the control group (*n* ≥ 20) (ns non-significant; ****p < 0.0001).

Developmental toxicity can be caused by various mechanisms, including interference of the compound with gene expression, cell growth and differentiation, homeostasis or inhibition of angiogenesis^47,48^. To investigate potential developmental and in vivo genotoxicity, zebrafish embryos were incubated in the presence of CYS from a very early developmental stage (1 day post-fertilization (dpf)) until most organs are fully developed (5 dpf). For the positive control 3,4-dichloroaniline^49^ various malformations including spinal curvature and edema were observed, which ultimately led to death. CYS-treated zebrafish embryos did not develop malformations with 100% survival until 5 dpf at their highest tested soluble concentration (20 µM) (Fig. 3a). Due to its higher solubility in the incubation medium, Cysto-180 was tested up to 200 µM, showing the same outcome. Thus, the maximum tolerated concentration of CYS in zebrafish embryos is above their solubility limit. In view of their potent antibacterial activity with MICs in the sub-µM range^15^, these data suggest that CYS might have a large therapeutic window.

Furthermore, it was reported that substances, which are cardiotoxic in humans, show the same effect in zebrafish embryos with a very high accuracy^50^. Especially human ether-à-go-go-related gene (hERG) channel inhibition is regarded as a major risk factor predictive of cardiotoxicity. Inhibition of this ion channel induces cardiac QT interval prolongation, resulting in a specific type of arrhythmia called torsades de pointes, which is associated with high mortality rates^21,51^. The phenotypical endpoints determined in the zebrafish embryo assay included heartbeat rate, cardiac rhythm and the development of pericardial edema^44^. Upon treatment with the known hERG-inhibitor terfenadine, embryos showed a significant, concentration-dependent increase in pericardial area (Fig. 3b, c). Additionally, examination of the heartbeat rate revealed a significant and concentration-dependent decrease of beats per minute with apparent arrhythmia in the vast majority of treated embryos (Fig. 3d). CYS-treated embryos neither showed pericardial malformations nor significant changes in pericardial area or heartbeat rate (Fig. 3b–d). These results suggest a favorable cardiotoxic safety profile of CYS in vivo.

The correlation between human and zebrafish toxicology was also observed for drug-induced hepatotoxicity. The assessment of in vivo hepatotoxicity offers the opportunity to identify not only the toxic effects of the parent molecule but also the potential toxicity of in vivo metabolites. At the same time, the actual in vivo distribution of a drug and its metabolites is considered, which can cause adverse effects if a compound shows accumulation in certain compartments, such as the liver. Functional toxicity, such as bile acid pump inhibition or steatosis, is also more effectively assessed in vivo than in vitro. For intoxicated zebrafish embryos, liver degradation can be easily observed by quantifying liver size reduction and observation of histological changes after treatment^45^.

Several mechanisms of drug-induced hepatotoxicity were proposed for the positive control valproic acid, namely formation of reactive metabolites, disturbance of the mitochondrial function and fatty acid metabolism and finally induction of oxidative stress^30^.

**Fig. 4:**
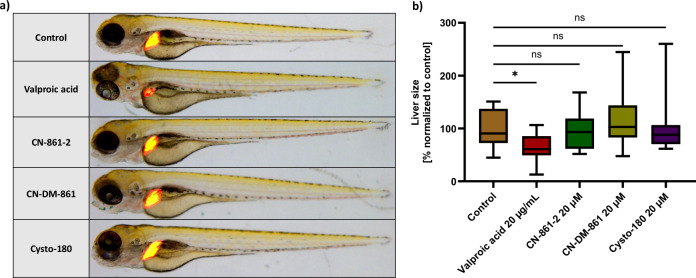
In vivo toxicity evaluation of CYS in zebrafish embryos revealed no abnormalities in liver morphology. **a** No in vivo hepatotoxic effect was observed for CYS-treated zebrafish embryos. Transgenic zebrafish embryos [Tg(fabp10a:DsRed; elaA:EGFP)] were checked for liver size reduction after treatment as endpoint readout. Liver degeneration was examined by comparing the fluorescent liver area to the control group. **b** CYS-treated embryos did not show a significant reduction in liver size, in contrast to the positive control valproic acid. Box plot (quartiles Q1 to Q3, including median) with whiskers (min to max) is shown for a quantitative representation of liver size. Statistical significance was analyzed by ordinary one-way ANOVA with multiple comparisons to the control group (*n* ≥ 15) (ns non-significant; *p < 0.05).

The transgenic fish line [Tg(fabp10a:DsRed; elaA:EGFP)] with fluorescent liver cells was used, facilitating the quantification of liver size^45^. Valproic acid-treated embryos showed significant liver size reduction, reflecting its hepatotoxicity. CYS did not show any significant reduction in liver size, thus suggesting an advantageous and non-hepatotoxic profile in vivo (Fig. 4a, b).

### Molecular off-target identification revealed SCARB1 as primary binding partner of CYS in eukaryotes

Phenotypical assays offer a comprehensive overview of potential intoxications; nevertheless, they frequently fail to provide insights into molecular mechanisms including binding partners of the investigated compounds.

For this purpose we designed and synthesized the CYS photo-probe Cysto-354 to investigate eukaryotic target proteins of CYS via affinity-based protein profiling (AfBPP) (Supplementary Figs. 7–27)^52^. Cysto-354 was tested for potential cytotoxicity, revealing a slight effect on cell viability at the highest assay concentration (37 µM) and no effect at any of the lower concentrations tested (≤12.3 µM) (Supplementary Fig. 28).

AfBPP aims to identify molecular binding partners via photo-reactive cross-linking followed by enrichment of the bound proteins. In order to exclude enriched but unspecifically bound proteins, competition of the binding site was performed by co-treatment with the unfunctionalized parent compound (here: CN-861-2). In such cases, specific binding is identified by reduction of enrichment in the presence of the parent compound.

**Fig. 5:**
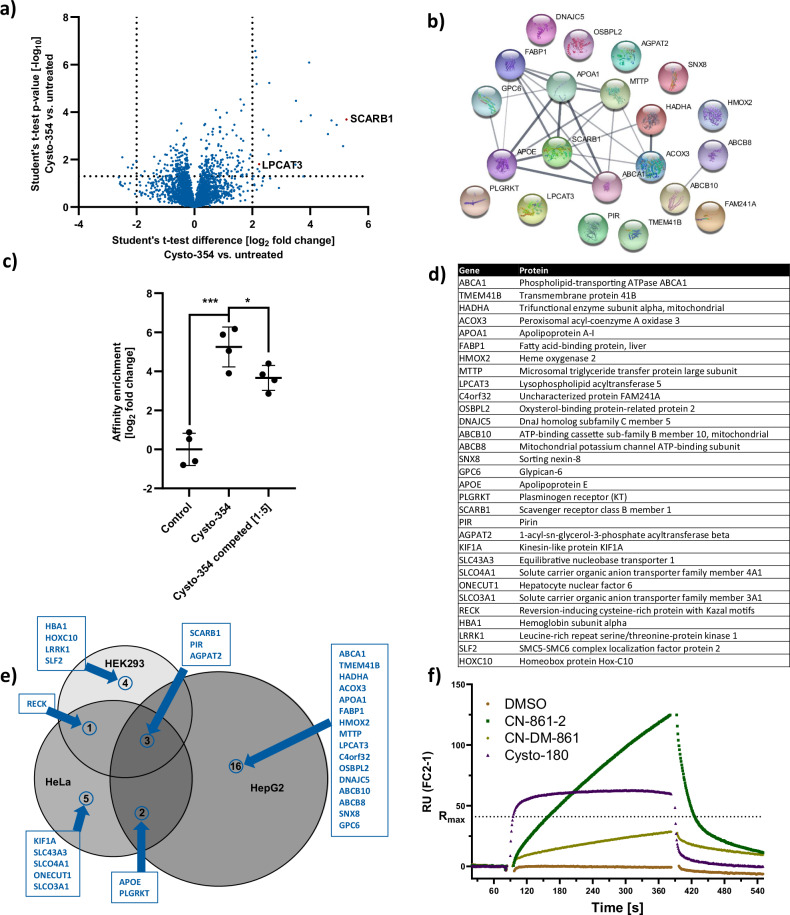
Molecular off-target identification revealed SCARB1 as primary binding partner of CYS in eukaryotes. **a** Affinity-based protein profiling (AfBPP) revealed significant enrichment of the SCARB1 protein. HepG2 cells were treated with the CYS photo-probe Cysto-354. After affinity enrichment, the protein abundances were compared to an untreated control (volcano plot). Significant enrichment was analyzed by two-tailed unpaired Student’s *t* test (cut-offs for enrichment: *p* [–log_10_] >1.3 and abundance difference [log_2_] >2) (*n* = 4). **b** STRING database analysis of significantly enriched proteins showed functional clustering with respect to cholesterol transfer activity due to lipoprotein and lipid binding (GO-term false discovery rate: 5.05e−7) (created using STRING db (version 12.0)). **c** SCARB1 showed the highest enrichment and significant competition upon CN-861-2 addition, indicating specific binding by CYS. Data are shown as mean ± standard deviation. Significant enrichment and competition were analyzed by two-tailed unpaired Student’s *t* test (*p < 0.05; ***p < 0.001) (*n* = 4). **d** List of significantly enriched proteins after Cysto-354 treatment in HepG2, HeLa-CCL-2 and HEK293. **e** Venn diagram of enriched proteins for CYS AfBPP in HeLa, HEK293 and HepG2 cells (created using BioVenn). **f** Binding analysis using SPR confirmed binding of CYS to the SCARB1 protein, exceedingthe theoretical maximal response (R_max_) of 41 RU (response units).

By performing the AfBPP assay with Cysto-354-treated HepG2 cells, an immortalized liver cell line which is widely used in early toxicity profiling of drug candidates, we were able to identify significantly enriched proteins, which belong to a functional cluster of cholesterol transfer activity due to lipoprotein and lipid binding (Fig. 5a, b). Proteins within this functional cluster had in common, that they interact with the highly enriched cholesterol-, lipid- and lipoprotein-receptor scavenger receptor class B member 1 (SCARB1).

By comparing significantly enriched and competed proteins, we were able to identify SCARB1 (Fig. 5c) and lysophosphatidylcholine acyltransferase 3 (LPCAT3) as potential specific binding partners of CYS. To gain insights across diverse cell types, this assay was replicated utilizing both HEK293 (kidney) and HeLa (cervix) cell lines. SCARB1 was observed to be enriched in all tested cell types, whereas LPCAT3 was not enriched in HEK293 or HeLa cells (Fig. 5d, e). The transcriptional coregulator pirin (PIR) and the acyltransferase AGPAT2 were observed to be enriched in all cell types (Fig. 5e), however, the proteins were not competed by CN-861-2 addition, indicating only unspecific binding of Cysto-354^53^. Noteworthy, human topoisomerase (TOP1, TOP2A, TOP2B) enrichment was not observed in the whole cell environment of HepG2, HeLa and HEK293, potentially explaining the lack of observable cellular (geno-)toxicity (Supplementary Fig. 29).

In conclusion, SCARB1 appears to be the primary off-target protein of CYS in eukaryotic cells. Biophysical binding analysis via surface plasmon resonance (SPR) (Biacore X100, Cytiva) was used to characterize and confirm the direct interaction of CYS and SCARB1. For this, biotinylated SCARB1 (Acrobiosystems) was immobilized on a SPR sensor chip, coated with streptavidin (sensor chip SA, Cytiva). All tested CYS derivatives (20 µM) showed interaction with the immobilized SCARB1 protein, exhibiting different binding kinetics (Fig. 5f). However, CYS appeared to not only bind specifically to SCARB1, as indicated in the competition assay, but we also observed some unspecific interactions in the assay characterized by exceeding the theoretical maximal response R_max_ (~41 RU) with increasing CYS contact time and concentration, which did not result in saturation. This effect is most likely mediated by the previously observed π-π-stacking of CYS’s aromatic systems, which occurs subsequent to initial specific binding^54^. Consequently, we were not able to determine the equilibrium dissociation constant (K_D_) of CYS to SCARB1 reliably.

Nevertheless, encouraged by this finding, we further characterized SCARB1 binding and functional inhibition with regard to a potential novel therapeutic indication of CYS.

### Binding of CYS to SCARB1 leads to functional inhibition of HCVpp entry into hepatocytes

The functional inhibition of SCARB1 was evaluated to examine the effects of CYS interaction with the protein.

SCARB1 binds different ligands such as phospholipids, cholesterol esters, lipoproteins, phosphatidylserine, but especially high-density lipoproteins (HDL) with high affinity. Thus, SCARB1 plays a dual role in cholesterol metabolism. Firstly, it promotes cholesterol efflux in peripheral tissues such as the arterial wall, aiding in the removal of excess cholesterol. Secondly, it serves as a substantial receptor for HDL cholesterol particles, facilitating the transfer of cholesterol, lipids and lipoproteins back into the liver^55–57^. Thus, it plays a crucial role in many physiological and pathophysiological processes including cholesterol and lipid homeostasis, cardiovascular disease, liver disease and cancer^58–60^. Importantly, SCARB1 also serves as a crucial entry receptor for the hepatitis C virus (HCV) into hepatocytes^61,62^.

**Fig. 6:**
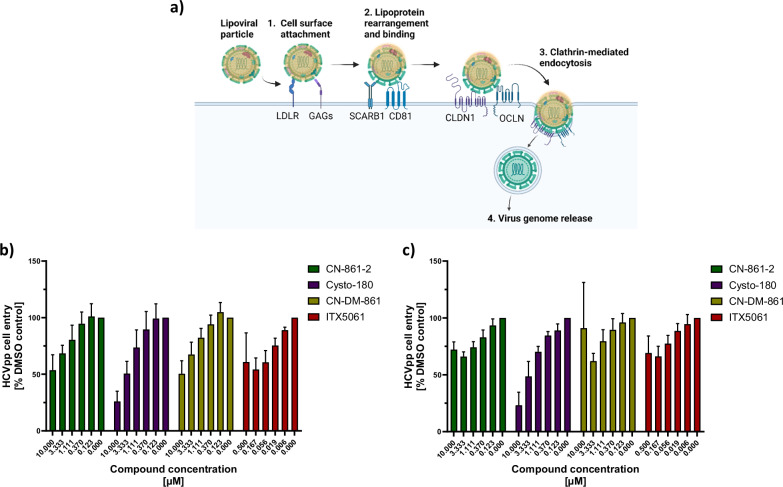
Binding of CYS to SCARB1 leads to functional inhibition of HCVpp entry into hepatocytes. **a** SCARB1 is known for its crucial role in binding and transfer of HCV lipoviral particles into hepatocytes (Created in BioRender. Risch, T. (2025) https://BioRender.com/z15x436). **b** HCVpp entry assays with glycoprotein JFH-1 (genotype 2a). Binding of CYS to SCARB1 leads to concentration-dependent inhibition of HCVpp entry into hepatocytes. ITX5061 was used as positive control. **c** HCVpp entry assays with glycoprotein Con1 (genotype 1b) also showed concentration-dependent entry inhibitory activity of CYS. Data are shown as mean values with standard deviation (*n* = 5).

Chronic hepatitis C is a viral infection that causes liver cirrhosis and hepatocellular carcinoma. In 2019, 1.5 million people were newly infected with the hepatitis C virus, with 290,000 infection-related deaths in the same year^63^. After infection, HCV circulates in the bloodstream as lipoviral particle and thereby gets in contact with the basolateral surface of hepatocytes to which it attaches by low-affinity interaction of lipoviral-associated ApoE to low-density lipoprotein (LDL) receptors and glycosaminoglycans (GAGs). Subsequently, SCARB1 binds the lipoviral-associated lipoproteins, initiating the lipid- and cholesterol-transfer activity of the protein, which probably mediates dissociation of the virus particle from its associated lipoproteins. Importantly, SCARB1 binds the HCV surface glycoprotein E2, causing a conformational change, which enables binding of E2 by CD81. Consequently, CD81 mediates lateral movement and interaction of HCV with claudin-1 (CLDN1), facilitating its endosomal cell entry and release of the viral genome (Fig. 6a)^64^.

We investigated the functional interaction of CYS with SCARB1 by examining the reduction of HCV pseudoparticle (HCVpp) cell entry into hepatocytes (Huh-7.5) upon CYS treatment. To this end, we used the lentivirus-based pseudotype assay, where the HCVpp was equipped with glycoproteins derived from two different genotypes (JFH1 from genotype 2a and Con1 from genotype 1b). The SCARB1-inhibitor ITX5061 was used as a positive control^60^. The luciferase reporter gene was used to quantify HCVpp entry. CYS treatment successfully inhibited HCVpp cell-entry in a concentration-dependent manner for both tested genotypes. CN-861-2 and CN-DM-861 exhibited similar inhibitory capacities, while Cysto-180 was the most effective derivative with 50% entry inhibition at a concentration of ~3.3 µM (Fig. 6b, c).

To elucidate the specificity of CYS in inhibiting SCARB1 as the entry receptor of HCVpp, we examined the effect of CYS on the SCARB1-independent entry of vesiculovirus pseudoparticles (VSVpp) equipped with respective viral glycoprotein. Indeed, we demonstrated that CYS exhibited no inhibitory effect on VSVpp cell entry (Supplementary Fig. 30). Hence, these results provide evidence for the specificity of functional inhibition of SCARB1 as a cause of CYS-mediated HCVpp cell entry inhibition.

Applying a bioinformatic molecular docking approach (MOE), only one potential CYS binding site within the modeled structure of SCARB1 was identified. The determined binding pocket is located at the extracellular domain of the protein, potentially important for interaction with native binding partners. CYS blocking of this site might hinder interaction of these binding partners with SCARB1, potentially explaining the inhibitory properties of CYS against HCVpp entry^65^. However, based on the docking results, multiple poses of CYS in the binding pocket appear feasible (Supplementary Fig. 31). Future studies will validate the mode of binding of CYS to SCARB1 in more detail and enable the opportunity for structure-guided optimization towards efficient inhibition of this host target.

These findings could not only broaden the potential therapeutic applications of CYS from a broad-spectrum antibiotic to an antiviral agent, but also extend to various non-anti-infective indications. The inhibition of SCARB1 by CYS might affect its lipid- and cholesterol transporter activity in vivo. In previous in vivo mouse studies, suppression of SCARB1 by ITX5061 successfully led to increased beneficial HDL cholesterol levels and partially reduced atherosclerotic lesions^60^. In addition, inhibition of SCARB1 is currently under investigation for potential therapeutic benefits across various medical conditions, including arteriosclerosis and other cardiovascular diseases, non-alcoholic fatty liver disease, or specific cancer types associated with the upregulation of SCARB1^58–60,66,67^. For ITX5061, safety in humans has been demonstrated^68,69^ and it might be concluded that modulation of cellular functions and lipid metabolism via inhibition of SCARB1 is not a particular concern. However, since the inhibition of SCARB1 by CYS is an early finding, future experiments will need to demonstrate CYS efficacy and safety in dedicated in vivo studies.

Herein, we demonstrated the safety of cystobactamids in cell culture assays and in in vivo zebrafish embryo models. The antibiotics showed slight uncoupling of the mitochondrial ETC, which might contribute to their ROS protective properties, while cystobactamids might additionally serve as direct radical scavengers based on their structural properties. This influence on the ETC does not seem to have harmful effects in vitro or in vivo as assessed by cell viability as well as developmental, cardio- and hepatotoxicity in zebrafish embryos. In vitro DMPK studies revealed glucuronidation and amide bond hydrolysis as the main biotransformation pathways of cystobactamids. Their metabolic stability was substantially enhanced by supplementation with cobicistat, an OATP- and CYP-inhibitor, offering the opportunity to explore combination therapy as a viable approach to improve PK/PD properties of cystobactamids in vivo. In addition, on a molecular level, cystobactamids bound to the cholesterol and lipoprotein receptor SCARB1 as the main eukaryotic off-target proteins in the whole cell environment. By binding to SCARB1, they actively prevented the entry of hepatitis C virus into hepatocytes. Analysis of the binding mode to SCARB1 might guide further development and optimization of cystobactamids, along with related compound classes such as albicidin and coralmycin, for various possible therapeutic areas.

## Methods

### Cell culture

All cell types were cultivated at 37 °C with 5% CO_2_. HepG2, HEK293, HCT116, CHO-K1, HeLa-CCL-2 and Huh-7.5 were purchased from ATCC. The respective cell media (Gibco) were supplemented with 10% FBS (Gibco) before usage. Cells were used between passages #5 and #30. To obtain biological repeats, cells were split separately for at least two passages.

### Topoisomerase IIα inhibition

Inhibition of topoisomerase activity was examined using the human topoisomerase II alpha decatenation assay kit (Inspiralis) as indicated by the manufacturer. Briefly, a compound dilution series was prepared in DMSO. Water, dilution buffer, assay buffer, plasmid and enzyme were mixed with the compound solution and incubated at 37 °C for 30 min. A positive control (100% activity) was prepared without the compound, and a negative control (0% activity) was prepared without the compound and the enzyme. The reaction was stopped by adding gSTEB-buffer and chloroform/isoamylalcohol (24:1 v/v). The samples were vortexed and centrifuged before running gel electrophoresis. The gel was stained with ethidium bromide and imaged using a Fusion Fx gel imager (Vilber Lourmat). Subsequent analysis was done using ImageJ and GraphPad Prism (Version 10.0.2).

### Cytotoxicity

Cells were washed with PBS and 0.5 mL trypsin was added. Afterwards, cells were incubated for 5 min before adding 10 mL medium containing 10% FBS. Per well, 120 µL cell suspension (5 × 10^4^ cells/mL) was seeded in transparent 96-well cell bind plates and incubated for 2 h (37 °C, 5% CO_2_). A dilution series (1:3) of test compounds was prepared in respective media containing 10% FBS and added (60 µL) to the prepared cell plate. Cells were incubated for a further 72 h (37 °C, 5% CO_2_). Next, 20 µL MTT (5 mg/mL in PBS) was added to each well and incubated for 2 h (37 °C, 5% CO_2_). The wells were emptied and 100 µL isopropanol/10 N HCl (1000:4) per well was added.

The plates were analyzed by measuring the absorbance at 570 nm (plate reader Infinite® 200 Pro, Tecan). After normalization of data to the respective solvent controls, the calculated percentage of growth inhibition was plotted using GraphPad Prism software (version 10.0.2).

### Minimum inhibitory concentration (MIC)

Cystobactamid MICs were determined using *E. coli* ATCC25922 following EUCAST guidelines for standard microbroth dilution in cation-adjusted Mueller-Hinton broth (MHBII). In a second experiment, MHBII was supplemented with 10% (v/v) FBS, reflecting conditions in cell culture experiments, and shifts in MIC were recorded.

### Micronucleus test

CHO-K1 cells were washed and seeded as described above with a concentration of 5 × 10^4^ cells/mL. Cells were treated with CYS (20 µM, 100 µM for Cysto-180) and incubated for 24 h. Mitomycin C (0.05 µM), doxorubicin (0.05 µM) and etoposide (0.25 µM) were used as positive controls. Cells were washed and stained with Hoechst (5 µg/mL) (Thermo) in F12 media for 15 min. Subsequently, cells were washed three times with PBS and imaged using Celldiscoverer 7 (Zeiss).

### Mitochondrial toxicity

Seahorse mitotox assay was carried out according to Agilent seahorse XF mito tox assay kit user guide (kit 103595-100, Agilent). Briefly, sensor cartridge was hydrated at 37 °C, non-CO_2_, one day before the assay started in 200 µL water and 2 h in calibrant solution before usage. HepG2 cells were seeded with a density of 10 × 10^3^ per well in 80 µL and incubated overnight at 37 °C, 5% CO_2_. On the day of the assay, 100 mL Seahorse XF DMEM medium was prepared by the addition of glucose (10 mM), pyruvate (1.0 mM) and glutamine (2.0 mM). CYS compound dilutions were prepared in assay medium as well as positive controls menadione (50 µM) and Rot/AA (1.0 µM). Cells were washed with assay medium and compounds were added, followed by 2 h incubation at 37 °C, non-CO_2_. Oligomycin (1.5 µM) and FCCP (1.0 µM) were prepared and added to the respective ports in the sensor cartridge. The utility plate with sensor cartridge was inserted into the Seahorse system (Agilent) and calibrated. Afterwards, the cell plate was added and measurement of the oxygen consumption rate (OCR) was started. The assay was repeated three times independently with at least 6 technical replicates per condition. Uncoupling MTI was calculated as follows:

1 $${\boldsymbol{Uncoupling\; MTI}}=\frac{{\boldsymbol{Max\; Oligo\; OCR}}({\boldsymbol{Compound}})-{\boldsymbol{Min\; Oligo\; OCR}}({\boldsymbol{Ve}}{\boldsymbol{h}}{\boldsymbol{icle}})}{{\boldsymbol{Max\; FCCP\; OCR}}\left({\boldsymbol{Ve}}{\boldsymbol{h}}{\boldsymbol{icle}}\right)-{\boldsymbol{MinOligo\; OCR}}({\boldsymbol{Ve}}{\boldsymbol{h}}{\boldsymbol{icle}})}$$

### Mitochondrial superoxide formation

CYS was tested for its potential to induce oxidative stress in U-2 OS cells. Therefore, 120 µL of a cell suspension in McCoy’s (+10% FBS) containing 5 × 10^4^ cells/mL were seeded into black 96-well imaging plate (BD Falcon). The plates were incubated for two days (37 °C, 5% CO_2_) until the cells reached approximately 70% confluence. Subsequently, the cells were washed with HBSS buffer, prior to adding CYS (20 µM) and the ROS inducer menadione (50 µM) as positive control, along with the staining solution (HBSS, calcium, magnesium, 5 µM MitoSOX red and 10 µg/mL Hoechst (Thermo)). To check for ROS protective properties, menadione (50 µM) was added directly to the staining solution. The cells were incubated for 1–2 h at 37 °C and 5% CO_2_. Afterwards, the cells were washed twice with HBSS. The cells were imaged using an automated fluorescence microscope (Celldiscoverer 7, Zeiss) (excitation: 485 nm, emission: 535 nm) to analyze the fluorescence intensity of the superoxide tracker MitoSOX red. The experiment was carried out with six replicates per condition. Quantification was done by high-content image analysis using the ZEN software (version 3.4, blue edition, Zeiss). Data presentation was done using GraphPad Prism (version 10.0.3) with ordinary one-way ANOVA including multiple comparisons to the control group for statistical analysis of the sample without ROS induction via menadione in the staining solution. Multiple unpaired *t* test was used to compare samples for ROS protective properties, which were incubated with menadione in the staining solution.

### Metabolic stability in mouse liver microsomes

For the evaluation of phase I metabolic stability, the compound (1 μM) was incubated with 0.5 mg/mL pooled mouse liver microsomes (Xenotech, Kansas City, USA), 2 mM NADPH, 10 mM MgCl_2_ at 37 °C for 120 min on a microplate shaker (Eppendorf, Hamburg, Germany). The metabolic stability of testosterone, verapamil and ketoconazole was determined in parallel to confirm the enzymatic activity of mouse liver microsomes. For combination experiments, cobicistat was added to the incubation mixture together with test compounds. The incubation was stopped after defined time points by precipitation of aliquots of enzymes with 2 volumes of cold internal standard solution (15 nM diphenhydramine in 10% methanol/acetonitrile). Samples were stored on ice until the end of the incubation and precipitated protein was removed by centrifugation (15 min, 4 °C, 4000 *g*). Concentration of the remaining test compound at the different time points was analyzed by HPLC-MS/MS (Vanquish Flex coupled to a TSQ Altis Plus, Thermo Fisher, Dreieich, Germany) and used to determine half-life (*t*_1/2_).

### Metabolic stability in mouse hepatocytes

For the evaluation of combined phase I and phase II metabolic stability, the compound (1 μM) was incubated with 0.25 × 10^6^ cells/mL of pooled mouse hepatocytes (Xenotech, Kansas City, USA). Cells were thawed in Leibovitz’s L-15 medium without phenol red (ThermoFisher Scientific, Waltham, USA). Briefly, cells were transferred into 50 mL of medium, followed by centrifugation at 55 *g* for 6 min. Supernatant was discarded, and the cell count was determined after gently resuspending the cell pellet in 1mL of medium. Hepatocytes were diluted to 0.5 × 10^6^ cells/mL and incubated at 37 °C, 700 rpm for 10 min, to achieve the desired final cell count after addition of an equal volume of test compounds in medium, leading to a final test concentration of 1 µM at 1% DMSO. Samples were incubated for 240min at 37 °C, 700rpm, and the incubation was stopped after defined time points by precipitation of aliquots in 4 volumes of cold internal standard solution (12.5nM diphenhydramine in 10% methanol/acetonitrile). For the determination of metabolism in the presence of cobicistat, this drug was added together with test compounds in the desired concentration range. The metabolic stability of testosterone, verapamil, ketoconazole and 7-hydroxycoumarine were determined in parallel to confirm the enzymatic activity of mouse hepatocytes. Samples were stored on ice until the end of the incubation and precipitated protein was removed by centrifugation (15 min, 4 °C, 4,000 g). Concentration of the remaining test compound at the different time points was analyzed by HPLC-MS/MS (Vanquish Flex coupled to a TSQ Altis Plus, Thermo Fisher, Dreieich, Germany) and used to determine half-life (t_1/2_). Intrinsic clearance was calculated as follows:

2 $${\boldsymbol{C}}{{\boldsymbol{L}}}_{{\boldsymbol{i}}{\boldsymbol{n}}{\boldsymbol{t}}\,}\,\left[\frac{{\rm{\mu }}{\bf{L}}}{{\boldsymbol{m}}{\boldsymbol{i}}{\boldsymbol{n}}{\times }{{\bf{10}}}^{{\bf{6}}}{\boldsymbol{c}}{\boldsymbol{e}}{\boldsymbol{l}}{\boldsymbol{l}}{\boldsymbol{s}}}\right]=\frac{{\boldsymbol{Incubation\; volume}}\left[{\rm{\mu }}{\boldsymbol{L}}\right]\times {\boldsymbol{ln}}({\bf{2}})}{{\boldsymbol{Cells\; in\; incubation\; mixture}}[{{\bf{10}}}^{{\bf{6}}}]\times {{\boldsymbol{t}}}_{{\bf{1}}/{\bf{2}}}}$$

For metabolite identification studies, test compounds were incubated at 10 µM final concentration and samples were analyzed using HPLC-HRMS (Ultimate 3000 coupled to a Q Exactive Focus, Thermo Fisher, Dreieich, Germany). LC conditions were as follows: column: Accucore Phenyl-Hexyl (2.6 µm, 100 x 2.1 mm; Thermo Fisher, Dreieich, Germany); temperature 40 °C; flow rate 0.500 mL/min; solvent A: water + 0.1% formic acid; solvent B: acetoni-trile + 0.1% formic acid; gradient^70^: 0–4.0 min 2–35% B, 4.0–7.0 min 35–98% B, 7.0–8.0 min 98% B, 8.0–10.0 min 2% B. MS analysis was performed using full scan mode (switching polarity, full MS resolution 35,000, scan range 200–2,000; data-dependent MS/MS (ddMS^2^) resolution 17,500, stepped collision energy with 17.5, 35, 52.5). Blank samples using DMSO were run in parallel for background subtraction. Sample processing for metabolite identification was performed using Compound Discoverer 3.2 (Thermo Fisher, Dreieich, Germany). Metabolites were identified based on mass shifts and feasibility of the metabolic reaction, also in view of MS peak intensities over time.

### Zebrafish handling

Handling of adult zebrafish and experiments with zebrafish embryos were performed in accordance with the EU directive 2010/63/EU and the German Animal Welfare Act (§11 Abs. 1 TierSchG). An automatic aquatic eco-system (PENTAIR, Apoka, UK) was used for zebrafish housing. Adult zebrafish were mated pairwise in our zebrafish facility after one night in the mating cages (light/dark cycle: 14 h/10 h). The eggs were collected after 2 h of mating and placed in a petri dish with fish water (pH 7.36 ± 0.08, conductivity: 800 ± 50 µs). The eggs were washed with 0.3x Danieau´s medium (17 mM NaCl, 0.2 mM KCl, 0.12 mM MgSO_4_, 0.18 mM Ca(NO_3_)_2_, 1.5 mM HEPES, pH 7.1–7.3, and 1.2 µM methylene blue). 300 eggs were placed per Petri dish and stored in the incubator at 28 °C overnight. 24 h post-mating, the unfertilized eggs were discarded. The 0.3x Danieau´s media in the petri dishes was replaced every 24 h and embryos with developmental issues were euthanized using ice water. Embryo-/genotoxicity, cardiotoxicity and hepatotoxicity were tested using zebrafish embryos younger than 120 h post-fertilization (hpf). The wild-type AB line was used for embryo-/genotoxicity and cardiotoxicity, and the transgenic line Tg(fabp10a:DsRed; elaA:EGFP) was used for hepatotoxic evaluation. Embryos were treated with phenylthiourea (PTU) starting from 1 day post-fertilization (dpf) for hepato- and cardiotoxicity analysis. Previous to compound addition, embryos were dechorionated using pronase (1 mg/mL) and washed several times. The embryos were euthanized on ice after a maximum of 120 hpf and frozen at −20 °C.

### Maximum tolerated concentration (MTC)

Zebrafish embryos (AB line) were dechorionated with pronase at 1 dpf. For embryo-/genotoxicity testing, 20 embryos per condition were treated with 20 µM CN-861-2, CN-DM-861 (solubility limit) and 200 µM (solubility limit), 100 µM, 50 µM and 20 µM Cysto-180 from 1 dpf till 5 dpf. DMSO (0.2%) was used as negative control and 3,4-dichloroaniline (16 µg/mL) as positive control. For acute in vivo toxicity testing, embryos were treated from 4 to 5 dpf accordingly with maximal soluble concentrations of CYS and DMSO (0.2%) and 3,4-dichloroaniline (32 and 64 µg/mL) as negative and positive controls, respectively. Kaplan-Meier curves were generated using GraphPad Prism (version 10.0.3, GraphPad, Boston, MA, USA). Dead embryos were defined when no heart beat was observable.

### In vivo cardiotoxicity in zebrafish embryos

Embryos were prepared as described above. At 2 dpf, the embryos were placed in 6-well plates with 10 embryos per well. Embryos were treated with CYS (20 µM) and the positive control terfenadin (10, 20 µM) in Danieau’s media. Danieau’s with DMSO was used as a negative control. At least 20 embryos were tested per condition. Embryos were incubated at 28 °C for 24 h. Afterwards, embryos were anesthetized with tricaine added to the media (40 µg/mL, 0.004% (*w/v*)) and videos were recorded using a stereomicroscope (1.25 × magnification) (Stemi 508, Zeiss) with Media Recorder (version 4.0) and analyzed via DanioScope software (version 1.2.208, Noldus). The embryos were checked with attention to specific cardiotoxic phenotypes defined as decreased heartrate, arrhythmia and pericardial edema. Death was defined as the absence of a heartbeat. Data presentation was done using GraphPad Prism (version 10.0.3) with ordinary one-way ANOVA including multiple comparison to the control group and multiple unpaired *t* test for statistical analysis.

### In vivo hepatotoxicity in zebrafish embryos

Zebrafish embryos [Tg(fabp10a:DsRed; elaA:EGFP)] were prepared as described above. Embryos were placed in 6-well plates with 10 embryos per well. Treatment with CYS as well as the positive control valproic acid (20, 40 µg/mL) was done by soaking from 3 to 5 dpf in 0.3x Danieau´s media. Per condition, at least 15 embryos were treated. Danieau´s media with DMSO was used as a negative control. The embryos were incubated at 28 °C. After 48 h treatment, the embryos were checked via a fluorescence stereomicroscope (M205 FA, Leica) with attention to liver degeneration. Fluorescence images were taken after anesthetizing the embryos with tricaine (40 µg/mL) and placing them laterally (excitation: 558 nm, emission 583 nm, 35× magnification). The specific phenotypic hepatotoxic endpoint was liver degeneration defined as liver size reduction. The liver size was calculated using ImageJ software (format: 8 bit, threshold: 40–255, binary, particle size: 10.0-infinite). The liver size of the control embryos was set to 100%. The relative liver sizes of treated embryos were calculated accordingly and normalized to the control group. Data presentation was done using GraphPad Prism (version 10.0.3) with ordinary one-way ANOVA including multiple comparison to the control group for statistical analysis.

### Affinity-based protein profiling (AfBPP) sample preparation

Cells were seeded in 100 mm cell-bind petri dishes with a concentration of 2.8 × 10^6^ cells per sample and incubated (37 °C, 5% CO_2_) for 3 days to reach ~80% confluence. Cells were washed with warm PBS prior to compound addition. Compound solutions as well as the DMSO control were generally added as freshly prepared solutions in pre-warmed, serum-free culture medium. Affinity-based proteome profiling was performed by treating the respective cell line with Cysto-354 (2.5 µM) for 3 h. For competition samples, cells were treated with CN-861-2 (12.5 µM) for 1 h, prior to addition of photo-probe Cysto-354 (2.5 µM) for 3 h incubation time. Control samples were treated with DMSO. Afterwards, cells were UV-irradiated for 10 min on ice, scraped off and transferred to Eppendorf tubes for washing with 1 mL cold PBS (centrifuge 5 min, 500*g*, 4 °C). The cell pellet was stored at −80 °C until lysis. Further sample preparation was done as previously described^13^. Briefly, the cell pellet was lysed in 0.4% SDS in PBS by sonication (Bandelin Sonoplus). After proteome adjustment (1000 µg/sample), azide-alkyne cycloaddition of labeled proteins with biotin was performed. Subsequently, the proteome was precipitated and washed with acetone and methanol, followed by avidin-bead enrichment. Afterwards, samples were digested (trypsin platinum, Promega), desalted and dried in a speedVac, before solubilizing the samples in 1% FA. Samples were filtered (Merck Millipore, UFC30GV0S) and transferred to HPLC autosampler vials (QuanRecovery, Waters).

### LC-MS measurement of HepG2 proteome

Peptides were measured and online-separated using an UltiMate 3000 nano HPLC system (*Dionex*) coupled to a *Bruker* timsTOF Pro mass spectrometer via a CaptiveSpray nano-electrospray ion source and *Sonation* column oven. Peptides were first loaded on the trap column (Acclaim PepMap 100 C18, 75 µm ID x 2 cm, 3 µm particle size, *Thermo Scientific*), washed with 0.1% formic acid in water for 7 min at 5 µL/min and subsequently transferred to the separation column (*IonOpticks* Aurora C18 column, 25 cm × 75 µm, 1.7 µm) and separated over a 60 min gradient from 5% to 28% B, then to 40% B over 13 min, followed by 10 min at 95% before re-equilibration and at a flow rate of 400 nL/min. The mobile phases A and B were 0.1% (v/v) formic acid in water and 0.1% (v/v) formic acid in acetonitrile, respectively. The timsTOF Pro was operated in data-dependent PASEF mode with the dual TIMS analyzer operating at equal accumulation and ramp times of 100 ms each with a set 1/K_0_ ion mobility range from 0.85 to 1.40 V × s × cm^-2^. The capillary voltage of the CaptiveSpray source was set to 1500 V. 10 PASEF scans per topN acquisition cycle were performed, resulting in a total cycle time of 1.17 s. The mass range was set from 100 to 1700 *m/z*. Only Precursors reaching an intensity threshold of 1750 arbitrary units were considered for fragmentation; precursors reaching a target intensity of 14500 arbitrary units were dynamically excluded for 0.4 min. The quadrupole isolation width was set to 2 *m/z* for *m/z* < 700 and to 3 *m/z* for *m/z* > 800. The collision energy was ramped linearly as a function of the mobility from 59 eV at 1/K_0_ = 1.6 V × s × cm^-2^ to 20 eV at 1/K_0_ = 0.6 V × s × cm^-2^. TIMS elution voltages were calibrated linearly to obtain the reduced ion mobility coefficients (1/K_0_) using three Agilent ESI-L Tuning Mix ions (*m/z* 622, 922 and 1,222) spiked on the CaptiveSpray Source inlet filter.

MS raw data was analyzed using MaxQuant software (version 2.0.3.0) and peptides were searched against Uniprot database for Homo sapiens (taxon identifier: 9606, downloaded on 11.03.2022, canonical, reviewed). Carbamidomethylation of cysteine was set as fixed modification and oxidation of methionine and acetylation of *N*-termini were set as variable modifications. Trypsin was set as a proteolytic enzyme with a maximum of 2 missed cleavages. For the main search, precursor mass tolerance was set to 4.5 ppm and fragment mass tolerance to 0.5 Da. Label-free quantification (LFQ) mode was activated with an LFQ minimum ratio count of 2. Second peptide identification was enabled, and false discovery rate (FDR) determination carried out by applying a decoy database and thresholds were set to 1% FDR at peptide-spectrum match and at protein levels and “match between runs” (0.7 min match and 20 min alignment time windows) option was enabled. Normalized LFQ intensities extracted from the MaxQuant result table proteinGroups.txt were further analyzed with Perseus software (version 2.03.1).

### LC-MS measurement of HeLa-CCL-2 and HEK293 proteome

Sample analysis was done as previously described^13^. Samples have been analyzed using nanoElute nano flow liquid chromatography system (Bruker, Germany) coupled to a timsTOF Pro (Bruker, Germany). Loading of the samples to the trap column (Thermo Trap Cartridge 5 mm) was performed, followed by washing with 6 µL 0.1% FA with a flow rate of 10 µL/min. Transferring of the peptide samples to the analytical column (Aurora Ultimate CSI 25 cm x 75 µm ID, 1.6 µm FSC C18, IonOpticks) was done, with subsequent separation by a gradient elution (eluent A: H2O + 0.1% FA, B: ACN + 0.1% FA; 0% to 3% in 1 min, 3% to 17% in 57 min, 17% to 25% in 21 min, 25% to 34% in 13 min, 34% to 85% in 1 min, 85% kept for 8 min) using a flow rate of 400 nL/min. A Captive Spray nanoESI source (Bruker, Germany) was applied for ionizing the peptides at 1.5 kV with 180 °C dry temperature at 3 L/min gas flow. timsTOF Pro (Bruker, Germany) was operated using default dia-PASEF long gradient mode with TIMS set to 1/K0 start at 0.6 V s/cm^2^, end at 1.6 V s/cm^2^, with a ramp and accumulation time of 100 ms each and a ramp rate of 9.43 Hz. Mass range was set from 100.0 Da to 1700 Da with positive ion polarity. Dia-PASEF mass range was arranged to 400.0 Da to 1201.0 Da with a mobility range of 0.60 1/K0 to 1.43 1/K0 and a cycle time of 1.80 s. Collision energy for 0.60 1/K0 was fixed to 20.00 eV and ramped for 1.6 1/K0 to 59.00 eV. Tuning MIX ES-TOF (Agilent) was applied for calibration of *m/z* and mobility. Raw data were processed using DIA-NN (version 1.8.1), and proteins were identified against Uniprot Homo sapiens reference proteome (Proteome ID: UP000005640, downloaded 27/12/2023). The default settings were used, except that the precursor charge range was from 2 to 4. C-carbamidomethylation was set as a fixed modification. To allow further data processing with Perseus Software, “--relaxed-prot-inf” was added in additional options. Further data analysis was performed in Perseus software (version 2.0.5.0), where the values were transformed to their log_2_-value and the biological replicates were grouped. To allow the comparison of the whole datasets, missing values were imputed by default settings and the differential protein abundance between different treatment regimens was evaluated using a two-tailed Student’s *t* test. The cut-offs for –log_10_ *P* were set to 1.3 (*P* = 0.05) and for t-test difference >2. Proteins fitting these thresholds were seen as significantly enriched compared to the control only treated with DMSO. Significant competition was evaluated by comparing CN-861-2 co-treated samples with Cysto-354-only treated samples^13^.

### Surface plasmon resonance (SPR)

Binding analysis of CYS to SCARB1 was done by using SPR (Biacore X100, Cytiva). Therefore, biotinylated SCARB1 (Acrobiosystems) was immobilized on a SPR sensor chip SA (Cytiva) on flow cell (FC) 2. Afterwards, CYS (20 µM) were solved and centrifuged in running buffer. CYS was tested by interaction analysis over the reference FC1 and FC2, where SCARB1 has been immobilized. To conclude binding, the subtraction of FC2-1 was analyzed. HBS-EP+ (Cytiva) with 1% DMSO was used as ra unning buffer.

### HCVpp cell entry assay

Lentiviral pseudotypes were prepared by 293T transfection as described previously^71^. 293T cells were seeded at a density of 3 × 10^6^ cells in 10 cm plates and incubated at 37 °C and 5% CO_2_. After 24 h, the cells were transfected with 2 µg of the lentiviral Gag-Pol expression construct pCMV-ΔR8.74^72^, 2 µg of the reporter plasmid coding for a firefly luciferase (pWPI-F-Luc-BLR)^73,74^ and 2 µg of either pcDNA3_CMV_dcE1E2_Con1, pcDNA3_CMV_dcE1E2_JFH^75^ or pczVSV-G^76^ coding for the glycoproteins of interest or 2 µg of the empty vector control (pcDNA3). For transfection, the plasmids were mixed in Opti-MEM containing a final concentration of 0.035 mg/ml polyethylenimine (PEI) and afterwards added to the cells. After overnight incubation, sodium butyrate was added at a final concentration of 10 mM. After 6 h of incubation, a medium change was performed and after a subsequent overnight incubation, the pseudoparticle containing supernatant was harvested and cleared of cell debris by passing through 0.45 µm pore size filter and used for entry assay.

The evaluation of the effect on entry by the compounds was performed on Huh-7.5 cells. Huh-7.5 cells were seeded in 96-well plates at a density of 7.2 x 10^3^/well and incubated for 24 h at 37 °C and 5% CO_2_. Compounds were diluted in six subsequent 3-fold dilutions with a final DMSO concentration of 1% and freshly harvested pseudoparticles were added and transferred to the cells. After 72 h of incubation, the cells were lysed with lysis buffer (1% triton-X-100, 25 mM glycylglycine, 15 mM MgSO_4_, 4 mM EGTA, 1 mM dithiothreitol) and frozen at -20 °C. The luciferase activity measurements were performed by transferring 72 µL of assay buffer (25 mM glycylglycine, 5 mM KPO_4_, 50 mM MgSO_4_, 10 mM EGTA, 2% ATP, 1 mM dithiotreitol) to a white 96-well plate and adding 20 µL of the cell-lysis suspension with subsequent adding of 40 µL D-Luciferin and immediate measuring using the Berthold LB960 Centro XS3 plate luminometer.

### Molecular docking

For molecular docking, the AlphaFold structure (AF-Q8WTV0-F1) of scavenger receptor class B member 1 was downloaded as a PDB file and loaded into MOE (version 2022.02). Structural issues were corrected using the “Structure Preparation” tool and the protein was protonated using “Protonate3D” with a set pH to 7.4. The structure was energy minimized before screening for a potential binding site with “Site Finder”. Atom dummies were loaded in the potential binding site. CYS derivatives Cysto-180, CN-861-2 and CN-DM-861 were prepared (protonated, energy minimized) in MOE and loaded into a compound database as a .mdb file. This .mdb file was used for docking the compounds to the previously prepared SCARB1 structure. Dummy atoms were selected as the target site with the triangle matcher (score: London dG) as the placement method and induced fit (score: GBVI/WSA dG) for refinement. Potential poses were browsed and optimized via protonation and energy minimization.

### Large language models (LLMs)

During the preparation of this work, the author(s) used ChatGPT (GPT-4) in order to polish the phrasing of some passages. After using this tool, the author(s) reviewed and edited the content as needed and take(s) full responsibility for the content of the publication.

## Supplementary information

Off-targets_Cystobactamids-SI_revised

## Data Availability

Datasets and further information of current and ongoing related studies are available upon request from the corresponding authors. The mass spectrometry proteomics data have been deposited to the ProteomeXchange Consortium via the PRIDE partner repository with the data set identifiers PXD053711 (HepG2) and PXD052895 (HEK293, HeLa).
